# Higher prevalence of rotavirus infection among out-born newborns transferred to a regional neonatal intensive care unit in Korea

**DOI:** 10.1186/s12887-022-03753-w

**Published:** 2022-11-29

**Authors:** Yoo-Jin Kim, Ji Hyuk Lee, Joon Kee Lee, Shin Ae Yoon, Sung-Il Woo

**Affiliations:** Department of Pediatrics, Chungbuk National University Hospital, Chungbuk National University College of Medicine, 1 Chungdae-ro, Seowon-gu, 28644 Cheongju, Korea

**Keywords:** Rotavirus, Newborn, Out-born, Neonatal intensive care unit, Nursery

## Abstract

**Background:**

Rotavirus is one of important pathogens which require infection control in nurseries and neonatal intensive care unit (NICU).

**Method:**

We retrospectively reviewed 1,135 out-born newborns who were transferred to a regional tertiary NICU of Chungbuk National University Hospital between January 2012 and December 2016. We assessed the clinical characteristics of newborns based on the results of rotavirus surveillance tests. The prevalence of rotavirus was evaluated according to the year, month, and season.

**Results:**

Among the 1,135 infants, 213 (18.8%) had positive results in the rotavirus surveillance test. The rotavirus positive group had a significantly higher gestational age, birth weight, and Apgar score. They also had a significantly higher rate of postpartum care centers when compared to the rotavirus negative group (45.5% vs. 12.6%, *P* < 0.001). Notably, the prevalence of rotavirus was significantly increased from 3.2 to 33.8% when infants were hospitalized 48 h after birth (*P* < 0.001). During the study period, there were no significant differences in the annual, monthly, or seasonal prevalence of rotavirus infection.

**Conclusion and discussion:**

These findings suggest that more active screening for rotavirus infection is necessary, especially for out-born newborns admitted to NICUs 48 h after birth or hospitalized after using postpartum care centers in Korea.

## Introduction

Rotavirus infections in newborns diversely manifest, ranging from asymptomatic to fatal necrotizing enterocolitis. Rotavirus is a pathogen that requires infection control owing to the potential for outbreaks in neonatal intensive care units (NICU) [1, 2]. Since 2007, the introduction of rotavirus vaccines has reduced the incidence of rotavirus enteritis in toddlers in Korea [3, 4]. However, newborns cannot avail these immunologic protective effects because of the inoculable age limit of rotavirus vaccines. Considering the recent reports of rotavirus associated white matter injury and its sequelae in newborns, infection control for rotavirus is critical in nurseries and NICUs [5–7].

Several studies have reported epidemiologic data of rotavirus, including outbreaks and variable positive rates of surveillance tests in nurseries and NICUs. The positive rate of surveillance tests in nurseries and NICUs varies from 13.3 to 31.7% and increases up to 43.7% during an outbreak [8–11]. Previous studies have reported that outbreaks are associated with in-hospital infections, with asymptomatic newborns considered to be the source of the outbreak as rotavirus has not been detected in the specimens collected from medical staff and environmental cultures [12, 13]. These findings suggest that the surveillance of symptomatic and asymptomatic newborns is imperative. However, previous studies have had limited data on the epidemiology of rotavirus outbreaks, including the prevalence of symptomatic newborns with jaundice, gastroenteritis, and seizures [1, 8–11, 14].

We also experienced two rotavirus epidemics in the NICU in 2016. Infection surveillance on medical staff and environmental cultures were all negative. We tried to establish a strategy for prevention of spreading of rotavirus in NICU. We hypothesized that an influx of out-born newborns whose rotavirus infection status is unknown leads to outbreak of rotavirus. In this study, we aimed to determine the prevalence and clinical manifestations of rotavirus infection in out-born newborns who were transferred to a regional NICU in Korea. Additionally, we compared the prevalence according to the age of the newborns upon admission and the annual, monthly, and seasonal prevalence of infection.

## Methods

### Study design and participants

We retrospectively reviewed the medical records of 1,135 out-born newborns who were admitted to Chungbuk National University Hospital NICU between January 2012 and December 2016, and for whom the results of initial rotavirus surveillance test at admission were available. Enrolled infants were transferred from local obstetrics and gynecology hospitals or admitted through outpatient clinics or emergency room. The data collection procedure was approved by the Institutional Review Board of Chungbuk National University Hospital (IRB No. 2020-04-20), and the requirement for informed consent was waived owing to the retrospective nature of the study. All methods were carried out in accordance with relevant guidelines and regulations.

During the study period, surveillance of rotavirus infection was conducted using fecal rotavirus antigen tests in all out-born newborns upon admission. The rotavirus antigen test was a sandwich-type enzyme immunoassay that used monoclonal antibodies against the most immunogenic rotavirus protein, VP6. RIDASCREEN® Rotavirus (R-Biopharm AG, Darmstadt, Germany) and Gemini (Stratec Biomedical AG, Birkenfeld, Germany) kits were used. The results of the rotavirus antigen tests were reported regularly, three times a week (Monday, Wednesday, and Friday). Standard cohort isolation in an incubator or isolated room for out-born newborns was maintained until the confirmation of a negative result.

### Measurements

We divided the infants according to the results of the rotavirus antigen tests and compared the clinical manifestations between the positive and negative groups. Clinical characteristics, including gestational age (GA), birth weight, sex, Apgar scores at 1 and 5 min, delivery mode, small for GA (birth weight < 10th percentile), premature rupture of membranes, pregnancy-induced hypertension, gestational diabetes mellitus, antenatal steroid use, use of postpartum care centers (Sanhujoriwon) [15], and age at NICU admission were analyzed. GA was determined based on the last maternal menstrual period and then modified using the Ballard test.

We reviewed the chief complaints of the infants and categorized the initial problems requiring NICU admission according to the following: neurologic, cardiorespiratory, gastrointestinal, genitourinary, metabolic, and hematologic disorders, skin infection, congenital anomaly, perinatal asphyxia, birth injury, low birth weight (birth weight < 2,500 g), and no perinatal care.

The chief complaints of out-born newborns were fever, jaundice, neurological disorders (seizure, hypotonia, and lethargy), cardiorespiratory disorders (apnea, tachypnea, cyanosis, bradycardia, tachycardia, and cardiac murmur), gastrointestinal disorders (vomiting, diarrhea, feeding difficulty, abdominal distension, hematemesis, hematochezia, and delayed defecation), infection, genitourinary problems (hematuria and hydronephrosis), metabolic disorders, and hematologic problems (petechia, anemia, and coagulopathy).

We compared the prevalence of rotavirus infection according to age upon NICU admission within 24 h, 25–48 h, and 48 h after birth, considering the 24–48 h incubation period of rotavirus [16]. We also investigated the annual, monthly, and seasonal prevalence of rotavirus infection. The seasons were categorized as winter (December–February), spring (March–May), summer (June–August), and autumn (September–November) [2, 3, 8].

### Statistical analysis

Continuous data was described as mean and standard deviation and was analyzed by Student’s t-test or one-way analysis of variance. Categorical variables are presented as percentages and frequencies and were compared using the chi-square or Fisher’s exact tests. All hypothesis tests were double-tailed, *P* < 0.05 was statistically significant. G*Power (version: 3.1.9.7) was used to calculate sample size and power value. SPSS version 25 (SPSS Inc., Chicago, IL, USA) was used for all statistical analyses.

## Results

### Clinical characteristics between both groups

During the study period, 1,135 infants were born in other hospitals and transferred to the tertiary NICU. Among them, 213 (18.8%) were positive in the rotavirus surveillance tests. The mean GA of the infants was 38 weeks, and the mean birth weight was 3,230 g. There was 620 boys and 515 girls. The rotavirus positive group had significantly higher GAs, birth weights, and Apgar scores. Additionally, the rate of postpartum care center use was significantly higher in the rotavirus positive group when compared to that in the negative group. In terms of age upon NICU admission, the rotavirus positive group was significantly older than the rotavirus negative group (negative group: 3.8 ± 4.8 days, positive group: 6.1 ± 3.3) (*P* < 0.001) (Table 1).

### Chief complaints between both groups

The chief complaints of the rotavirus negative group were cardiorespiratory diseases (45.2%), contrastingly, the chief complaints of the rotavirus positive group were fever (28.2%), jaundice (21.6%), gastrointestinal diseases (18.8%), and cardiorespiratory diseases (11.3%). Congenital anomalies, birth asphyxia, and cardiorespiratory diseases were significantly higher in the rotavirus negative group than in the positive group. In the rotavirus positive group, fever and central nervous and gastrointestinal system diseases were significantly higher (Table 2).

**Table 1:** Infant demographics

	Rotavirus negative group (*n* = 922)	Rotavirus positive group (*n* = 213)	*P-*value
Gestational age (weeks)	38.4 ± 1.5 (29^+ 5^–41^+ 5^)	38.7 ± 1.3 (34^+ 0^–41^+ 5^)	0.002
Birth weight (g)	3,215.7 ± 488.7 (1,550–4,920)	3,291.6 ± 498.1 (1,475–5,010)	0.042
Male	510 (55.3)	110 (51.6)	0.360
Apgar score at 1 min	8.5 ± 1.4	8.8 ± 0.7	< 0.001
Apgar score at 5 min	9.6 ± 1.3	9.8 ± 0.5	< 0.001
Cesarean section	335 (36.3)	64 (30.0)	0.069
Small for gestational age	27 (2.9)	4 (1.9)	0.491
Premature rupture of membrane	65 (7.0)	18 (8.5)	0.841
Pregnancy induced hypertension	8 (0.8)	0 (0.0)	0.394
Gestational diabetes mellitus	27 (2.9)	3 (1.4)	0.459
Antenatal steroids	0 (0.0)	1 (0.5)	0.093
Use of postpartum care center	116 (12.6)	97 (45.5)	< 0.001
Age at admission (day)	3.8 ± 4.8 (1–29)	6.1 ± 3.3 (1–22)	< 0.001

Values are presented as means ± SD (range) or n (%)

**Table 2:** The chief complaints of the enrolled infants

	Rotavirus negative group (*n* = 922)	Rotavirus positive group (*n* = 213)	*P-* value
Anomaly	51 (5.5)	7 (3.3)	0.180
Perinatal Asphyxia	17 (1.8)	0 (0.0)	0.046
Birth injury	6 (0.7)	1 (0.5)	0.761
Cardiorespiratory	420 (45.6)	25 (11.7)	< 0.001
Low birth weight	13 (1.3)	1 (0.5)	0.262
Neurologic	41 (4.4)	17 (8.0)	0.035
Fever	82 (8.9)	64 (30.0)	< 0.001
Infection	19 (2.1)	3 (1.4)	0.534
Gastrointestinal	62 (6.7)	45 (21.1)	< 0.001
Jaundice	179 (19.4)	47 (22.1)	0.382
Genitourinary	3 (0.3)	1 (0.5)	0.749
Metabolic	5 (0.5)	0 (0.0)	0.281
Hematologic	7 (0.8)	1 (0.5)	0.649
No perinatal care	18 (2.0)	1 (0.5)	0.128

Values are presented as n (%)

### Prevalence of rotavirus according to age at NICU admission

According to the age at NICU admission, the prevalence of rotavirus was 2.6% (11/416 infants) within 24 h of birth, 4.9% (7/143 infants) at 25–48 h, and 33.8% (195/576 infants) succeeding 48 h after of birth. The prevalence of rotavirus was significantly higher for NICU admissions admitted succeeding 48 h after birth than for within 48 h (*P* < 0.001). In a sub-analysis of 576 infants transferred succeeding 48 h after birth, there was no statistical difference between the two groups in GA, birth weight, and Apgar score. However, there was a significant difference in age at NICU admission (rotavirus negative group: 7.4 ± 5.7, rotavirus positive group: 6.6 ± 3.1) (*P* = 0.028). The use of postpartum care centers was also significantly different among the groups, with 29.1% (111/381) prevalence in the rotavirus negative group and 49.7% (97/195) prevalence in the rotavirus positive group (*P* < 0.001).

### Annual, monthly, and seasonal prevalence of rotavirus

**Fig. 1:**
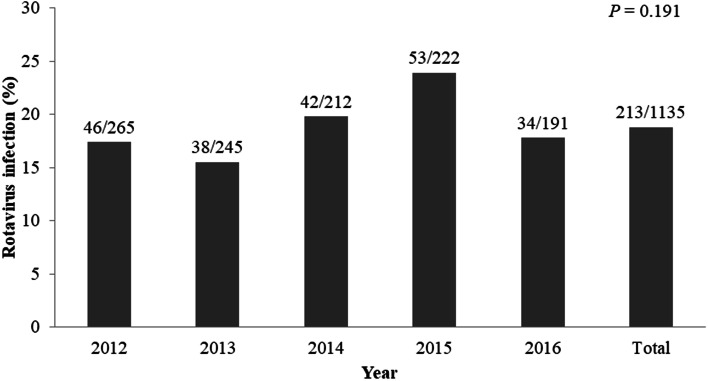
The annual prevalence of rotavirus infection among the study group

**Fig. 2:**
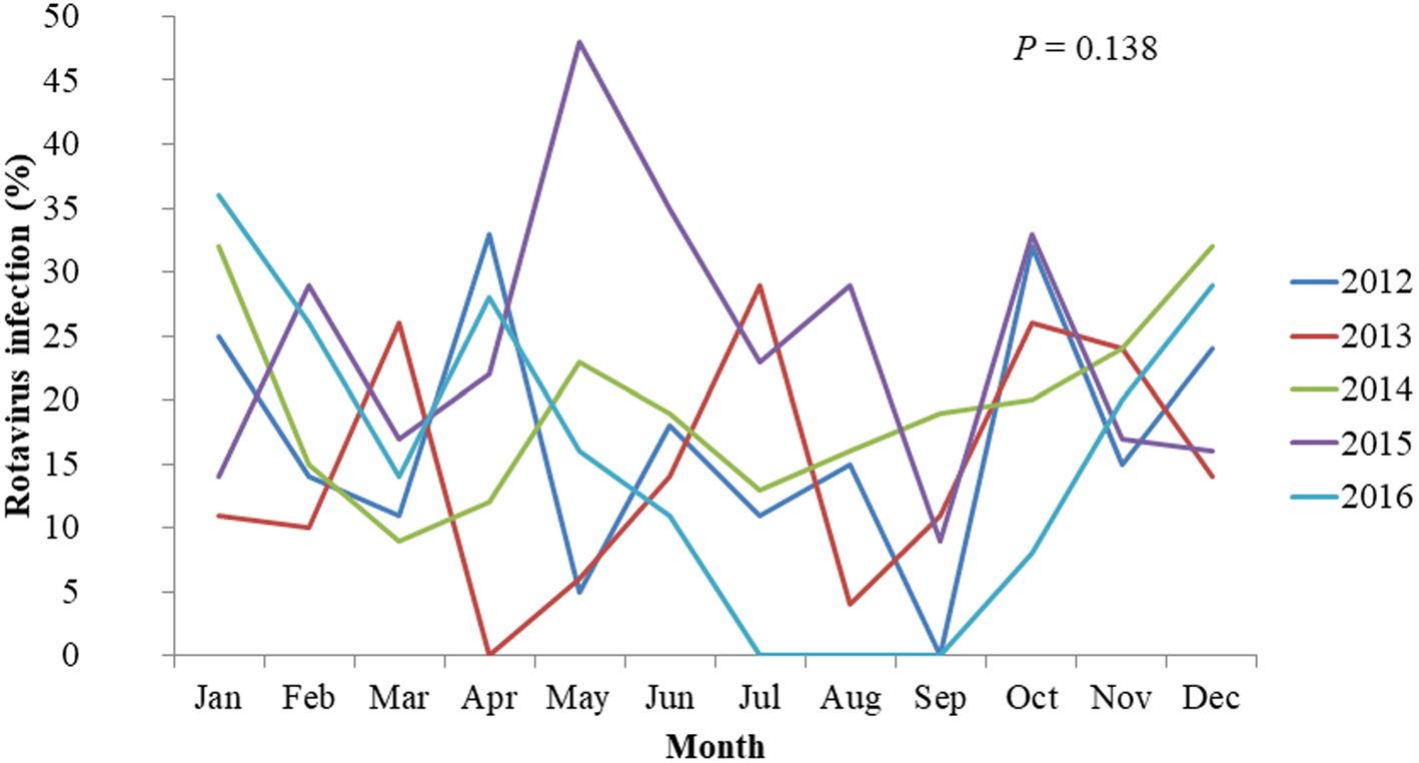
The monthly prevalence of rotavirus infection among the study group

From 2012 to 2016, there was no significant difference in the annual prevalence of rotavirus (*P* = 0.191) (Fig. 1), and there was no significant difference in the monthly prevalence of rotavirus (*P* = 0.138) (Fig. 2). Seasonal rotavirus positive rates were 22.0% (64/291) in winter, 19.0% (56/294) in spring, 15.8% (44/278) in summer, and 18.0% (49/272) in autumn (*P* = 0.298).

## Discussion

During the study period, the rotavirus positive rate was 18.8% and the rotavirus positive group showed significantly higher GAs, birth weights, Apgar scores, age at admission and postpartum care center use. The prevalence of rotavirus infection was higher in infants admitted to the NICU after 48 h of life. Among infants who admitted to the NICU 48 h after birth, postpartum care center use was associated with rotavirus positive results. The prevalence of rotavirus in this study was similar to that in previous studies by Kim et al. (13.3%) and by Shim et al. (25.2%) in other province of Korea [8, 9]. Other study by Moon et al. carried in another province of Korea showed higher positive rate of 31.4% with the antigen test but 11.7% with the polymerase chain reaction test [10]. Previous studies had short study period of 1 year. In this study, we observed no difference annual prevalence of rotavirus infection. There was no difference in the incidence rate according to month, year, or season, suggesting that the constant temperature and humidity environment of newborn rooms and postpartum care centers enables rotavirus to infect all year round [8, 12].

The GA, birth weights, and Apgar scores of the rotavirus negative group were significantly lower than those of the rotavirus positive group. The most common chief complaint of the rotavirus negative group was cardiorespiratory diseases originating from failure to transfer from fetal to neonatal adaptation immediately after birth. The rotavirus positive rate increased from 3.2% in infants hospitalized within 48 h of birth to 33.8% in newborns hospitalized succeeding 48 h after birth. This was the effect of the incubation period of rotavirus. In the comparison between newborns hospitalized succeeding 48 h after birth, the later hospitalizations among the rotavirus negative group was a result of the delay in the recognition of symptoms that require medical treatment as many of these infants were taken home and not cared for in medical centers. The rotavirus antigen test was positive for 18 infants who were transferred within 48 h of birth suggesting that monitoring rotavirus infection in out-born newborns is important, regardless of the time of hospitalization. Half of the rotavirus positive group (n = 97) had a history of postpartum center care. Of them, eight infants were hospitalized in the NICU after discharge from a postpartum care center and only two of them had siblings; therefore, the possibility of contacting rotavirus infection from sibling interaction was very low among this group. Consequently, thorough infection monitoring is needed for newborns with a history of postpartum center care.

The chief complaints of the transferred infants varied, and the symptoms of the rotavirus positive group were fever, jaundice, and gastrointestinal system issues. Fever is known to be the most common symptom of rotavirus infection in newborns [8, 9, 13]. A recent study in Korea reported that rotavirus infection is frequently observed in newborns hospitalized for neonatal jaundice [14]. Rotavirus is the main neonatal gastroenteritis causing pathogen, and in this study, 40.4% of infants hospitalized for gastrointestinal system disease were rotavirus positive. Rotavirus is known to cause systemic reactions of the central nervous and respiratory systems in addition to the gastrointestinal tract [17, 18]. In the rotavirus positive group, 17 infants hospitalized for seizures performed brain magnetic resonance imaging, but no white matter damage related to rotavirus infection was detected. In the rotavirus positive group, 24 infants with cardiorespiratory diseases underwent a respiratory virus polymerase chain reaction test to rule out the possibility of respiratory virus co-infection [19]. Only 2 of the 24 infants had a positive result for respiratory virus co-infection.

In nurseries and NICUs, rotavirus infection can cause significant complications from mild gastroenteritis to irreversible brain white matter damage. It can even cause death from necrotizing enteritis in premature infants who are hospitalized for long periods. Therefore, infection monitoring of asymptomatic out-born newborns is important as they can be the source of infection, making monitoring a critical part of infection control. In the rotavirus positive group, the utilization rate of postpartum care centers was significantly high suggesting the possibility of in-institute rotavirus outbreaks. This highlights the importance of rotavirus infection control in non-medical institutions, such as postpartum care centers in Korea. Early detection of asymptomatic newborns with rotavirus infection will be a clue for strategies to prevent rotavirus outbreaks in nurseries and NICUs. Therefore, we have to introduce rapid antigen kit for rotavirus screening at admission and take more attention to out-born newborns who transferred after postpartum care center use. At the time of discharge, infants who will use postpartum care centers after discharge should consider rotavirus screening.

This study has some limitations. First, as this was a retrospective study, accurate genotyping of the rotavirus positive group could not be performed. Therefore, it was not possible to determine the epidemiological relationship between infants born in the same obstetrics and gynecology departments or hospitalized simultaneously. A previous domestic study showed that rotavirus G4P is the most common genotype found in infants, so it is assumed that this genotype was among the infections in this study [9, 20]. Second, stool tests for mothers of positive newborns hospitalized within 48 h of birth were not performed, meaning vertical infection could not be excluded. However, previous studies have shown that the possibility of vertical infection of rotavirus is low; therefore, in-hospital/institute infection after birth is considered to be the main source of infection [8, 21]. Third, breastfeeding is known to reduce the positive rate of rotavirus infection and the severity of symptoms; however, this study did not investigate this effect on infection rates [22]. Additionally, in the case of newborns born in the Chungbuk National University Hospital, rotavirus tests were only performed when rotavirus symptoms were suspected; therefore, the rotavirus positive rate of inborn newborns in the hospital could not be compared. Since this study included only out-born newborns who required admission to the regional NICU, we could not compare the prevalence of rotavirus in healthy newborns in nurseries. Enrolled infants were sick and might be more susceptible to rotavirus infection. And this study was limited by its single-center and retrospective design, it is hard to generalize. However, the present study involved a large number of enrolled infants and conducted in the only tertiary NICU in a province in Korea. Although postpartum health care centers are a unique social culture in Korea, outbreaks of rotavirus and respiratory syncytial virus in nurseries and NICUs are often reported in other countries, such as China and India [1, 21, 23, 24]. Our data suggests that the influx of asymptomatic out-born newborns is a potential source for rotavirus outbreaks, again reinforcing the importance of monitoring rotavirus infection in these newborns as a key part of infection control.

## Conclusion

In this study, the authors investigated the rotavirus positive rate among out-born newborns admitted to an NICU in Korea. A high rotavirus positive rate was recorded in infants who were admitted to the NICU succeeding 48 h after birth or had a history of postpartum center care. These findings could be translated to establish the infection surveillance strategies in NICU including more active screening of rotavirus infection in out-born newborns. Outbreaks of rotavirus can be prevented through early detection of asymptomatic infants as a main approach to infection control.

## Data Availability

The datasets generated and/or analyzed during the current study are not publicly available due reason why data are not public but are available from the corresponding author (dalen@chungbuk.ac.kr) on reasonable request.

## Abbreviations

GA: Gestational age
NICU: Neonatal intensive care units
